# Structure, Functions, and Physiological Roles of the Lipocalin α_1_-Microglobulin (A1M)

**DOI:** 10.3389/fphys.2021.645650

**Published:** 2021-03-03

**Authors:** Jesper Bergwik, Amanda Kristiansson, Maria Allhorn, Magnus Gram, Bo Åkerström

**Affiliations:** ^1^Department of Clinical Sciences, Section for Infection Medicine, Lund University, Lund, Sweden; ^2^Division of Hematology and Transfusion Medicine, Department of Laboratory Medicine, Lund University, Lund, Sweden; ^3^Department of Clinical Sciences, Pediatrics, Lund University, Lund, Sweden

**Keywords:** antioxidant, reduction, heme, radicals, thiol, preeclampsia, acute kidney injury, radioprotection

## Abstract

α_1_-microglobulin (A1M) is found in all vertebrates including humans. A1M was, together with retinol-binding protein and β-lactoglobulin, one of the three original lipocalins when the family first was proposed in 1985. A1M is described as an antioxidant and tissue cleaning protein with reductase, heme- and radical-binding activities. These biochemical properties are driven by a strongly electronegative surface-exposed thiol group, C34, on loop 1 of the open end of the lipocalin barrel. A1M has been shown to have protective effects *in vitro* and *in vivo* in cell-, organ-, and animal models of oxidative stress-related medical conditions. The gene coding for A1M is unique among lipocalins since it is flanked downstream by four exons coding for another non-lipocalin protein, bikunin, and is consequently named α_1_-microglobulin-bikunin precursor gene (*AMBP*). The precursor is cleaved in the Golgi, and A1M and bikunin are secreted from the cell separately. Recent publications have suggested novel physiological roles of A1M in regulation of endoplasmic reticulum activities and erythrocyte homeostasis. This review summarizes the present knowledge of the structure and functions of the lipocalin A1M and presents a current model of its biological role(s).

## Introduction

Proteins of the lipocalin family are found in all but one branch of life, and 15–20 different proteins of this family have been confirmed and studied in the human body. The lipocalins share a common fold and are characterized by their ability to bind small, most commonly hydrophobic, molecules (Schiefner and Skerra, 2015). A multitude of different biochemical and enzymatic properties have been described for the lipocalins and they are involved in a large variety of biological functions, including immunoregulation, signal transduction, smell reception, tissue development, storage and transportation of molecules, and cell homeostasis. One of the members of the lipocalin family is α_1_-microglobulin (A1M), a protein that has been described as a tissue housekeeping protein responsible for removal of and protection against harmful oxidants and reparation of macromolecules. A1M was discovered over 45 years ago as a yellow-brown protein purified from human urine (Ekström et al., 1975), and it has been shown to be conserved in all studied vertebrate species. Following synthesis in the liver, it is secreted into the circulation and it is subsequently equilibrated over the vessel wall between the intra- and extravascular compartments of all organs. Early reports proposed an immunoregulatory role of A1M (Babiker-Mohamed et al., 1990a; Åkerström et al., 2000), but more recent reports have presented data of enzymatic reductase activity as well as heme- and radical binding functions, which reconceptualized the biological role to a housekeeping protein responsible for cleaning of tissues and antioxidant protection (Åkerström and Gram, 2014). The aim of this review is to give an overview of the structure, mechanistic functions and life cycle of A1M, and to put these into a physiological context.

## Structure

**FIGURE 1:**
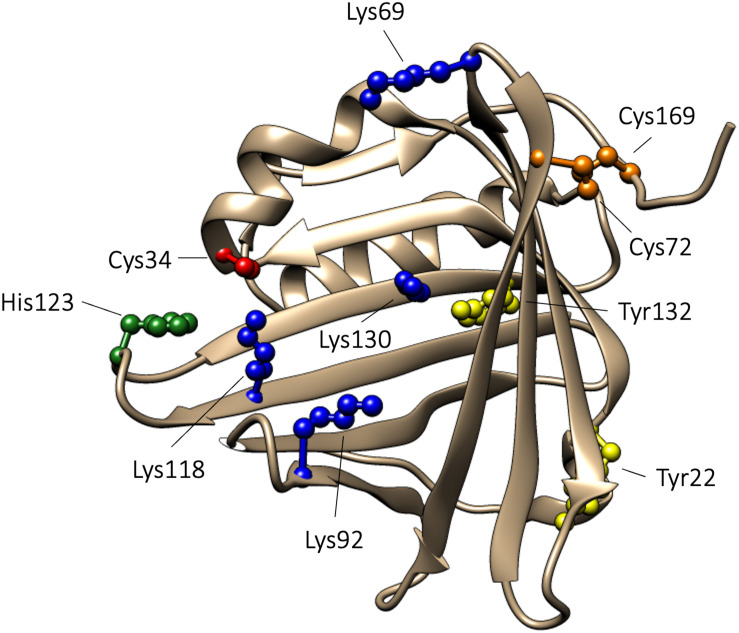
3D-structure of A1M. The 3D-structure of A1M is based on the published crystal structure (Meining and Skerra, 2012). Functionally important amino acids are highlighted: C34, described to be important for reduction, radical scavenging, and heme binding, is marked with red. Lysine residues (K69, K92, K118, and K130), described to be related to the yellow-brown modifications, are marked with blue. H123, suggested to take part in coordination of heme-iron, is marked with green. Tyrosine residues (Y22 and Y132), shown to be modified in radical scavenging reactions, are marked in yellow. The disulfide bridge, formed between C72 and C169, is marked in orange.

The proteins in the lipocalin family share a similar tertiary structure, which resembles a bucket. The bucket is formed by eight antiparallel β-strands shaped into a β-barrel with one open and one closed end. The lipocalin polypeptides contain 150–190 amino acids and are composed of a single polypeptide chain. Commonly, the lipocalins contain a binding site within the β-barrel where small hydrophobic compounds are bound (Flower et al., 2000). The crystal structure of A1M displays the characteristic lipocalin fold with a β-barrel and four loops at the open end of the bucket (Meining and Skerra, 2012). A1M has a disulfide bridge between C72 and C169, and a free thiol on the unpaired C34. C34 is located on one of the loops, loop 1, close to the open end of the bucket. It has been shown that C34 participates in one-electron oxidation and reduction reactions (Allhorn et al., 2005) and is involved in binding and neutralization of target compounds (Åkerström et al., 2007). Human A1M has a molecular weight of 26 kDa and is composed of a single polypeptide with 183 amino acids (Takagi et al., 1981; López Otin et al., 1984; Kaumeyer et al., 1986). A1M is a glycoprotein, which carries three different oligosaccharides (Amoresano et al., 2000) that can be found in different glycoforms. Two N-linked sialylated glycans are present on A1M, a biantennary glycan attached to N17 and a biantennary or triantennary glycan attached to N96. Additionally, O-linked structures, consisting of HexHexNAc glycans, are attached to T5 (Amoresano et al., 2000). A1M isolated from urine has been shown to be covalently modified with “chromophores,” suggested to be the result of binding of free radicals and/or heme degradation products. Covalent modifications have been shown to be located on C34, K69, K92, K118, and K130, and these chromophores contribute to the heterogeneous charge and yellow-brown color of urinary A1M (Escribano et al., 1991; Åkerström et al., 1995; Berggård et al., 1999a). Additionally, *in vitro* experiments using the synthetical radical 2,2’-azino-bis(3-ethylbenzothiazoline-6-sulfonic acid) (ABTS) have identified radical adducts on Y22 and Y132 (Åkerström et al., 2007). Finally, A1M has been described to contain two heme binding sites with different affinities for heme (Karnaukhova et al., 2014). Using molecular simulation, one heme binding site was found to be located buried in the lipocalin pocket in the vicinity of K92, K118 and K130, and the other binding site was located closer to the surface involving C34 and H123 (Rutardottir et al., 2016). The 3D structure of A1M, with important residues highlighted, is shown in Figure 1.

## Gene

The gene encoding A1M is denoted the α_1_-microglobulin-bikunin precursor gene (*AMBP*), and besides A1M it encodes a second protein, bikunin (Kaumeyer et al., 1986; Vetr and Gebhard, 1990). Bikunin is a structural component of the extracellular matrix and a Kunitz-type plasma proteinase inhibitor (Chen et al., 1992; Shigetomi et al., 2010). The *AMBP* gene is composed of 10 exons, where A1M is encoded by exons 1–6 and bikunin by exons 7–10. Additionally, exon 1 encodes a signaling peptide (Vetr and Gebhard, 1990). Transcription of the *AMBP* gene produces the AMBP protein, which is constituted by A1M and bikunin linked together by the tripeptide VRR (Lindqvist et al., 1992). Folding of the AMBP protein occurs in the endoplasmic reticulum (ER), from where it is further transported to the Golgi apparatus and post-translationally modified through the attachment of a chondroitin sulfate chain to the N-terminal part of bikunin. Subsequently, after leaving the Golgi apparatus, heavy chains (HC) are covalently bound to the chondroitin sulfate chain (Enghild et al., 1991). Three different kinds of HC exist in humans: HC1, HC2, and HC3. Attachment of HC1 and HC2 results in the formation of inter-α-trypsin inhibitor (IαI), whereas attachment of HC3 generates pre-α-inhibitor (PαI) (Fries and Blom, 2000). Before being secreted from the cell, the AMBP protein is proteolytically cleaved between A1M and bikunin. After secretion, no physical or functional association between A1M and bikunin, or their complexes, has so far been described. Consequently, the reason for co-synthesis of the two proteins is largely unknown, although recent evidence suggests that the presence of A1M is important for correct synthesis and post-translational modification of bikunin (Bergwik et al., 2020).

Transcription of the *AMBP* gene in the liver is regulated by hepatocyte nuclear factors (HNF 1–4) (Rouet et al., 1992, 1995, 1998). The expression of A1M is, similar to other antioxidation proteins, also regulated by the Keap1/Nrf2 system (Chorley et al., 2012; Campbell et al., 2013). Upregulation of the A1M expression has been found during oxidative stress related conditions induced by heme, hydrogen peroxide (H_2_O_2_), hemoglobin (Hb) and hydroxyl radicals (OH^•^) in primary skin keratinocytes, human cell lines and in skin and retinal explants (Olsson et al., 2007, 2011; Åkerström et al., 2017; Kristiansson et al., 2020b). Increased A1M concentrations in plasma samples have also been described in clinical conditions associated with oxidative stress. Women with preeclampsia (PE), a pregnancy related condition, displayed increased plasma A1M concentrations, as well as a correlation between plasma A1M and Hb concentrations, and between A1M and markers of oxidative stress, i.e., plasma peroxidation capacity and the amount of protein carbonyl groups (Olsson et al., 2010a). In that study, an upregulated A1M expression, i.e., increased A1M mRNA levels, was also found in placentas from the preeclamptic women.

The *AMBP* gene has been mapped to the lipocalin gene cluster in the 9q-32-33 region in man (Diarra-Mehrpour et al., 1989) and on chromosome 4 in mice (Salier et al., 1992). Intron number 6, located between exon 6 and 7, is very large and contains A/T-rich regions and retroposon elements. This suggests that intron 6 is an unstable region and a recombinatorial hotspot, supporting the theory that the A1M and bikunin genes are assembled from two ancestral genes (Lindqvist et al., 1999). The genetic construction of the *AMBP* gene is conserved in all species studied, which is covered in more detail below.

## α_1_-Microglobulin in Different Species

**FIGURE 2:**
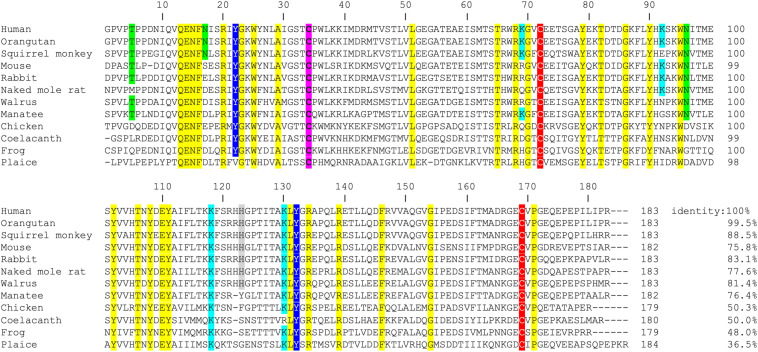
Amino acid sequence alignment of A1M from 12 different species. The amino acid sequence of human wild-type (wt) A1M and 11 additional species were retrieved from public data bases (www.ncbi.nlm.nih.gov and www.uniprot.org) and aligned using the http://www.ebi.ac.uk/Tools/msa/clustalw2/ software. The degree of identity of the different sequences to the human sequence is presented as percent relative to human A1M. Amino acids with reported functional impact in human A1M are marked: the C34, described to be important for reduction and antioxidant properties as well as heme binding, is marked with pink. Glycosylated positions (T5, N17, and N96) are marked with green. Lysine residues (K69, K92, K118, and K130), described to be related to the yellow-brown modifications, are marked with light blue. The disulfide bridge, formed between C72 and C169, is marked in red. H123, suggested to take part in coordination of heme-iron, is marked with gray. Tyrosines (Y22 and Y132), shown to be modified in radical scavenging reactions are marked in dark blue. Of these functionally important amino acids, five are completely conserved in all 12 species (C34, C72, K118, Y132, and C169). Additional 28 amino acids, which are identical between all species in the set, are marked with yellow. Human, *Homo sapiens*; Orangutan, *Pongo abelii*; Squirrel monkey, *Saimiri boliviensis boliviensis*; Mouse, *Mus musculus*; Rabbit, *Oryctolagus cuniculus*; Naked mole rat, *Heterocephalus glaber*; Walrus, *Odobenus rosmarus divergens*; Manatee, *Trichechus manatus latirostris*; Chicken, *Gallus gallus*; Coelacanth, *Latimeria chalumnae*; Frog, *Xenopus laevis*; and Plaice, *Pleuronectes platessa*.

α_1_-microglobulin has been found in a wide range of vertebrate species. The A1M protein has been detected, identified and purified from mammals (Åkerström and Berggård, 1979; Vincent et al., 1983; Åkerström, 1985; Åkerström et al., 1987; Tavakkol, 1991; Chan and Salier, 1993; Ide et al., 1994; Lindqvist and Åkerström, 1996; Nakata et al., 2011) amphibians (Kawahara et al., 1997), fish (Hanley and Powell, 1994; Lindqvist and Åkerström, 1999) and birds (Åkerström, 1985). The amino acid sequences from more than 60 species are available in public databases^1, 2^. Interestingly, the *AMBP* gene construction, i.e., the co-expression with bikunin, is preserved in all species where the *AMBP* gene has been described. Most of the published A1M-sequences (>50) contain several of the A1M-specific functional groups described above, i.e., the free thiol group of C34, the targets for covalent modifications K92, K118, K130, Y22, and Y132, the suggested heme binding residue H123 and the disulfide bridge C72-C169 (Figure 2). The A1M homolog sequences also contain the lipocalin motifs (SCR1, 2, 3), proposed to be essential for the lipocalin structural folding (Flower, 1996), whereas the predicted carbohydrate binding sites at position T5, N17, and N96 in human A1M (Escribano et al., 1990) are less conserved.

## Synthesis, Biodistribution, and Degradation

**FIGURE 3:**
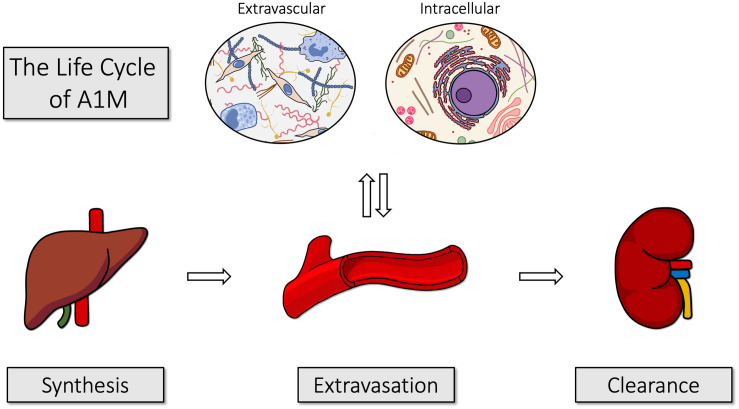
The life cycle of A1M. A1M is primarily synthesized in the liver, from where it is secreted into the blood. It is rapidly equilibrated over the vessel walls of all organs and can be taken up intracellularly. After exerting its functions, it is transported to the kidneys and filtrated in the glomeruli. Finally, it is reabsorbed by the proximal tubular cells where it is degraded together with any bound heme groups and/or radicals.

Figure 3 illustrates the life cycle of A1M, from its synthesis in liver, transport via blood to the extravascular space of all organs, and degradation in kidneys. The major site of synthesis is the liver (Tejler et al., 1978; Åkerström and Landin, 1985; Åkerström et al., 1995; Olsson et al., 2007). The protein is secreted from the liver to the blood, where the total concentration of A1M is approximately 2 μM (DeMars et al., 1989). In humans, about 50% of A1M in plasma is complex-bound to IgA via a reduction-resistant disulfide bond, 7% to albumin and 1% to prothrombin (Grubb et al., 1983; Berggård et al., 1997). Complex formation with other plasma proteins has been demonstrated in several other species. In rat, A1M was found to be covalently linked to fibronectin (Falkenberg et al., 1994) and α_1_-inhibitor-3 (Falkenberg et al., 1990), a homolog to the human protease inhibitor α_2_-macroglobulin. In plaice, high-molecular weight A1M-complexes have also been described (Lindqvist and Åkerström, 1999). Thus, the complex-forming propensity seems to be conserved from fish to mammals. Both free A1M and the complexed forms are rapidly equilibrated between the intra- and extravascular compartments and their half-lives in blood were determined to approximately 2–3 min in rats and mice (Wester et al., 2000; Larsson et al., 2001).

Due to its small size, free A1M is almost freely filtrated through the glomerular membranes out to the primary urine, where most of it is reabsorbed by the proximal tubular cells and catabolized (Nordberg et al., 2007). Some A1M, however, is excreted in the urine and the A1M-concentration in urine is a clinically used indicator of tubular renal damage (Ekström and Berggård, 1977; Nordberg et al., 2007).

A1M mRNA has been detected in most other human cell types besides the liver, *c.f.* kidney (Kastern et al., 1986; Leaver et al., 1994), placenta (Olsson et al., 2010a), stomach (Tavakkol, 1991), pancreas (Itoh et al., 1996; Berggård et al., 1998), skin (Olsson et al., 2011), retina (Cederlund et al., 2013) and blood cells (Leaver et al., 1994; Olsson et al., 2007). The A1M protein has been identified in the extravascular space of most organs, where it is associated to perivascular basal membranes and connective tissue (Ødum and Nielsen, 1994; Berggård et al., 1998), and especially abundant in the epidermis of skin (Bouic et al., 1985; Allhorn et al., 2003; Olsson et al., 2011) and intestinal/colon epithelium (Bouic et al., 1984; Larsson et al., 2001). It is often co-localized with elastin and collagen (Bouic et al., 1985; Ødum and Nielsen, 1994; Olsson et al., 2011) and has been shown to bind to collagen *in vitro* (Santin and Cannas, 1999; Olsson et al., 2011). Such a distribution of A1M in the extravascular space and extracellular matrix allows the protein to execute its protective functions (see below) mainly outside the blood circulation, at interfaces between the cells and the ambient environment (blood/tissue, air/tissue, intestinal lumen/villi), as well as at the interface between maternal blood and fetal tissues in placenta (Berggård et al., 1999b; May et al., 2011).

## Cell Binding, Uptake and Interaction With Mitochondria

Cell surface binding of A1M has been reported for a variety of cells, including peripheral lymphocytes (Fernandez-Luna et al., 1988; Babiker-Mohamed et al., 1990b; Wester et al., 1998, 2000), neutrophils (Méndez et al., 1986), erythrocytes (Kristiansson et al., 2020a), blood cell lines (Olsson et al., 2007, 2008), and keratinocytes (Olsson et al., 2011). The cell surface binding of A1M is specific and saturable, suggesting the presence of an A1M-receptor on the cells. The dissociation constant for binding of A1M on human T cells and mouse peripheral lymphocytes was estimated to approximately 10^–5^ M (Babiker-Mohamed et al., 1990b; Wester et al., 1998), but was slightly higher on the U937 histiocyte cell line, 10^–7^ M (Fernandez-Luna et al., 1988). However, up to date, a specific A1M-receptor has not yet been identified.

The erythroid cell line K562 (Olsson et al., 2008), erythrocytes (Kristiansson et al., 2020a), the hepatoma cell line HepG2 (Olsson et al., 2007), and primary keratinocytes (Allhorn et al., 2003) have been shown to internalize A1M from the culture medium. Intracellularly, A1M was shown to be localized to the mitochondria, and specifically to Complex I of the respiratory chain (Olsson et al., 2013). Mitochondrial localization, and an uptake of exogenously added A1M to mitochondrial Complex I of skin keratinocytes, blood cells and liver was shown by immunofluorescence, fluorescence-activated cell-sorting, and electron microscopy. Furthermore, mitochondrial A1M could be purified and identified by mass spectrometry. The functional role of A1M in the mitochondria is not fully understood, but since the cellular uptake of the protein is strongly intensified during apoptosis, it may be involved in assisting the mitochondria to maintain its energy delivery during apoptosis and cell death. A1M may also, at the same time, counteract and eliminate the ROS generated by the mitochondrial respiration to prevent oxidative damage to surrounding healthy tissue. As a result, it was suggested that A1M has a role in maintaining mitochondrial redox homeostasis (Olsson et al., 2013).

## Molecular Mechanisms

**FIGURE 4:**
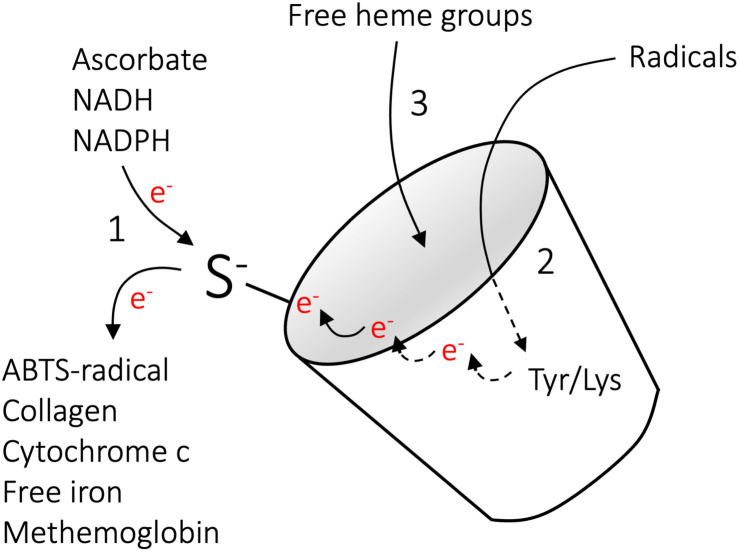
Molecular mechanisms of A1M. The molecular mechanisms implicated in the actions of A1M include (1) reductase activity (2) radical scavenging and (3) heme binding. The reductase activity is driven by the free thiol group of C34, is catalytic in the presence of the electron-donating co-factors NADH, NADPH, or ascorbate, and several substrates have been identified (Allhorn et al., 2005). The radical-scavenging reaction is also driven by C34, which, in the absence of electron-donating co-factors, can oxidize Y32 and Y132 to tyrosyl-radicals via intramolecular electron-transfer. This is followed by reactions with external radicals, ultimately leading to covalent trapping of the radicals and modification of the side-groups (Åkerström et al., 2007). *In vivo*, covalent modifications have been shown on the side groups of K69, K92, K118, and K130, suggesting a similar radical trapping mechanism on lysine residues. Binding of heme-groups were shown on two separate binding sites (Allhorn et al., 2002; Siebel et al., 2012; Rutardottir et al., 2016). Reproduced from Gunnarsson et al. (2017).

Comprehensive studies of the A1M protein over the last decades have revealed that A1M has a role as a physiological cell- and tissue protective antioxidant, and as described above three molecular mechanisms contribute to its function. These are reductase activity, radical scavenging and heme binding (Åkerström and Gram, 2014; Gunnarsson et al., 2017; Kristiansson et al., 2020c), and are summarized briefly below and schematically outlined in Figure 4.

### Reductase Activity

Owing to the free cysteine residue in position 34, A1M has the capacity to reduce several biological substrates, including metHb, cytochrome c, oxidized collagen I, oxidized low density lipoprotein, free iron and the synthetic radical ABTS (Figure 4, reaction 1). In addition to the thiol group of cysteine 34, the reductase activity appears to be dependent on three lysyl residues in position 92, 118, and 130 (Allhorn et al., 2005). The reduction potential of A1M has been observed to be catalytic in the presence of strong electron-donators such as nicotinamide adenine dinucleotide (NADH), nicotinamide adenine dinucleotide phosphate (NADPH) and ascorbate. However, since it has been shown that A1M can reduce physiological substrates both intra- and extracellular it is likely that there are additional physiologically electron-donating co-factors (Olsson et al., 2008, 2011; Rutardottir et al., 2013).

### Radical Scavenging

As shown with the synthetic radical ABTS, A1M retain the capacity to react with and “trap” radicals in a reaction that involves the reducing potential of C34 (Figure 4, reaction 2) (Åkerström et al., 2007). It has been demonstrated that 8–9 ABTS radicals are consumed per A1M molecule, with reduction of 5–6 ABTS radicals and covalent trapping of 2–3 radicals. The trapped ABTS radicals are localized to at least two different tyrosine residues, Y22 and Y132, confirming that each A1M molecule can covalently trap several ABTS molecules. As described above, a number of modifications of A1M side chains have been found on A1M isolated from urine (Åkerström et al., 1995; Berggård et al., 1999a; Sala et al., 2004), suggesting that the radical-trapping mechanism of A1M also operates *in vivo*. In addition, a recent report identified plasma conjugates between A1M and infused cancer-targeting drug compounds, and the authors speculated that the free thiol of C34 on A1M is involved in adduct formation (Su et al., 2019). Based on the observation that both the radicals and the A1M protein are electroneutral after the above-described reactions and consequently do no longer constitute an oxidative threat to tissues, cells and proteins, etc., the term “radical sink” was proposed (Åkerström et al., 2007; Åkerström and Gram, 2014).

### Heme Binding

α_1_-microglobulin binds heme with a K_d_ = 10^–6^ M and at a molar ratio of 2:1, such that two heme groups are bound to each A1M molecule (Figure 4, reaction 3) (Allhorn et al., 2002; Larsson et al., 2004; Siebel et al., 2012). The heme-binding property of A1M is evolutionarily conserved, and has been demonstrated for human, mouse, rat, guinea pig, cow, chicken and plaice A1M (Larsson et al., 2004). In addition to binding heme, A1M has been described to react with lysed red blood cells, purified Hb or the heme-containing enzyme myeloperoxidase (MPO). In this process, a truncated form of A1M, denoted t-A1M, was described to be formed (Allhorn et al., 2002; Cederlund et al., 2015). Interestingly, this processed form of A1M, lacking the four most C-terminal amino acids leucine-isoleucine-proline-arginine, was shown to be capable of degrading the heme group into a heterogeneous chromophore associated with the protein. A1M has been shown to bind and degrade heme both in its free monomeric form and when complex bound to IgA (Allhorn et al., 2002; Larsson et al., 2004).

## Association With Diseases

Based on its antioxidant functions, A1M has been proposed as a therapeutic agent in diseases where free radicals and heme groups are involved in the pathology, such as preeclampsia (Gunnarsson et al., 2017) and hemolytic conditions (Kristiansson et al., 2020c). A modified recombinant version of A1M named ROSgard^®^ is currently being evaluated in phase I clinical trials as a therapeutic agent against acute kidney injury (AKI) after open chest cardiac surgery. ROSgard is developed by the company Guard Therapeutics International. The use of A1M in therapy has been evaluated in a number of animal models and the studies are described below and summarized in Table 1.

**TABLE 1:** Summary of animal studies performed to examine the therapeutic potential of A1M against oxidative stress related diseases.

Species	Disease	Pathology	Treatment/route of admin	Effect of treatment	References
Rat	AKI	Infusion of HbF Increased glomerular permeability	A1M *i.v.* infusion 22.4 μg/min	Restored glomerular permeability	Sverrisson et al., 2014
Mouse	AKI	PRRT-induced kidney damage, DNA damage, upregulation of apoptotic- and stress-related genes, proteinuria, kidney lesions and death	A1M *i.v.* injection 7 mg/kg body weight, single dose	Decreased DNA damage and upregulation of apoptotic- and stress-related genes Reduced proteinuria and kidney lesions Less animal deaths	Kristiansson et al., 2019
Mouse	AKI	Rhabdomyolysis-induced mild AKI Upregulation of stress genes HO-1 and Hsp70 in kidneys	A1M *i.v.* injection 7 mg/kg body weight, single dose	Reversed upregulation of stress genes	Åkerström et al., 2019
Rabbit	IVH	IVH induced by glycerol injection Structural tissue and mitochondrial damage Expression of pro-inflammatory genes	A1M intracerebroventricular injection 0.235 mg, single dose	Decreased structural tissue and mitochondrial damage Reduced expression of pro-inflammatory genes	Romantsik et al., 2019
Sheep	PE	PE-like symptoms after starvation Structural damage to placenta and kidneys Increased glomerular permeability	A1M *i.v.* injection 1.8 mg/kg, two doses	Decreased structural damage to placenta and kidneys Restored glomerular permeability	Wester-Rosenlöf et al., 2014
Rabbit	PE	PE-like symptoms after HbF infusion Proteinuria and increased glomerular filtration Structural damage to placenta and kidneys	A1M *i.v.* injection 6 mg/kg, five doses	Reversed proteinuria and structural changes to placenta and kidneys Restored glomerular permeability	Nääv et al., 2015
Mouse	PE	PE-like symptoms (STOX1 transgenic) Gestational hypertension, proteinuria and organ alterations	A1M *i.p.* injection 0.27 mg, six doses	Decreased gestational hypertension Lowered hypoxia and nitrative stress in placenta Reduced cellular damage to placenta and kidneys	Erlandsson et al., 2019

### Kidney Damage

Kidney damage can occur through a multitude of different external or internal processes. As a result of a relatively high level of A1M in the kidneys, the use of it as a kidney protector in various conditions and as a biomarker for tubular damage (Yu et al., 1983; Nordberg et al., 2007) has gained attention. In fact, in a number of different animal models of renal damage, A1M has been observed to counteract or significantly reduce the renal damage. This includes (i) a study in rats where infused fetal Hb (HbF) induced increased glomerular permeability, (ii) a study in rabbits where renal damage was induced by infusion of HbF, (iii) a mouse study infusing radiopeptides, and (iv) a mouse rhabdomyolysis study where glycerol-injections triggered mild renal damage. In these studies, A1M was observed to reduce the damage-induced renal upregulation of stress genes and restore the compromised renal function (Sverrisson et al., 2014; Nääv et al., 2015; Åkerström et al., 2019; Kristiansson et al., 2019). Taken together, these studies suggest a protective effect of the kidneys by A1M from a variety of insults inducing oxidative stress-related injuries.

### Hemolysis

Hemolysis, i.e., the rupture of red blood cells, results in Hb- and heme-induced toxicity on cells and tissues and it is associated with a wide range of diseases. A1M has been shown to have erythroprotective effects (Kristiansson et al., 2020a), which could be a potential role *in vivo*, but may also offer a therapeutic opportunity in certain conditions [reviewed in Kristiansson et al. (2020c)]. Intraventricular hemorrhage (IVH) of prematurely born infants, is a severe hemolysis condition where fragile vessels in the brain rupture, leading to accumulation of toxic metabolites, such as free heme and Hb within the brain (Ley et al., 2016; Agyemang et al., 2017). In a preterm rabbit pup model of IVH, intracerebroventricular administration of A1M displayed a strong co-localization with Hb and resulted in a decreased structural damage and a normalized/reduced expression of pro-inflammatory genes (Romantsik et al., 2019).

### Preeclampsia

Preeclampsia is a pregnancy related disease associated with high blood pressure and albuminuria as defining clinical features (Young et al., 2010). The disease has been described to be associated with elevated concentrations of HbF in the maternal blood plasma during the second and third trimester, and in the fetus and placenta at term, and it was suggested that oxidative stress induced by the HbF-iron is critically involved in development of PE (Centlow et al., 2008; Olsson et al., 2010a; Anderson et al., 2011). Since oxidative stress, Hb and free heme are targets of the protective activities of A1M, it was suggested that A1M may be used as a therapeutic agent in PE (Gunnarsson et al., 2017). Further support for this notion is that human A1M plasma levels were observed to be increased and the expression of the gene was upregulated in liver and placenta of PE patients, suggesting that the protein is involved in the natural protection against PE (Olsson et al., 2010a; Anderson et al., 2011, 2016). In order to study the therapeutic effect of A1M, three different animal models of PE have been evaluated. First, starvation of pregnant ewes resulted in development of PE-like structural damages to both placenta and kidneys (Wester-Rosenlöf et al., 2014). Second, infusion of HbF in pregnant rabbits resulted in PE-like symptoms, i.e., proteinuria and an increased glomerular sieving coefficient as well as intra- and extracellular tissue damage in the kidneys (Nääv et al., 2015). Third, overexpression of the transcription factor Storkhead box 1 (STOX1) led to PE symptoms in pregnant mice, including hypertension, proteinuria and structural impairments of kidneys and placenta (Erlandsson et al., 2019). In these three studies, administration of A1M reduced structural and functional injuries closely linked to the PE pathology.

## Proposed Biological Roles

**FIGURE 5:**
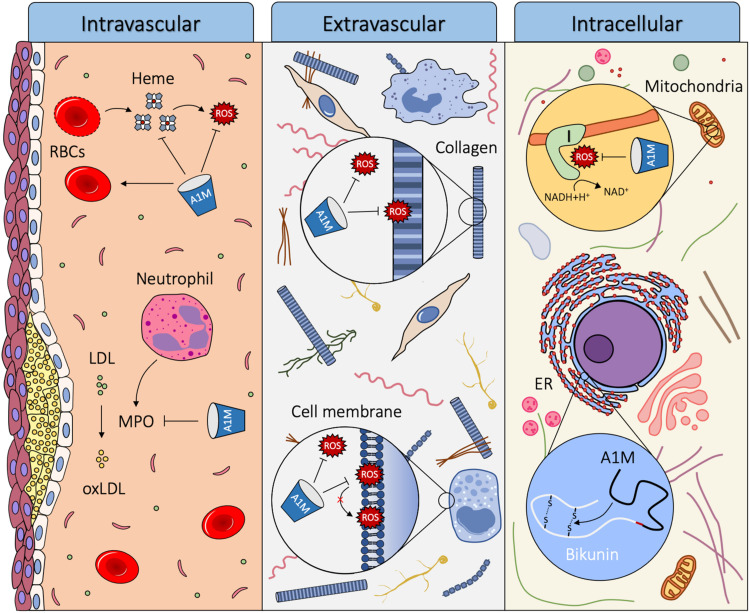
Proposed biological roles of A1M. Using its heme binding, radical scavenging and reductase activity, A1M can act as a housekeeping protein in three different compartments of the body: intravascular, extravascular and intracellular. Intravascularly, A1M acts a stabilizer of red blood cells, it reduces damage from hemolytic events by binding heme and reducing Hb and it inhibits oxidation of LDL by MPO. Extravascularly, A1M protects tissues, cells and macromolecules from oxidative insults, including reducing oxidation products formed on ECM structures and lipid peroxidation of cell membranes. Intracellularly, A1M is bound to Complex I of the respiratory chain in the mitochondria where it may have a radical scavenging function and preserves ATP-production. A1M also acts as a chaperone during the folding of bikunin in the ER.

α_1_-microglobulin is primarily produced in the liver and secreted into the circulation. From the blood, it is rapidly transported across the capillary walls of all organs, and equilibrated between intra- and extravascular compartments, from where it also can be internalized by cells. A1M has the ability to reduce oxidation products, scavenge radicals and bind free heme molecules, which indicate a role as a housekeeping protein that contributes to the antioxidation defense and detoxification and repair of oxidized macromolecules (Åkerström and Gram, 2014). A1M is thought to exert its function in the three different compartments described: intravascular, extravascular and intracellular. The proposed biological roles of A1M are summarized in Figure 5.

### Intravascular

Although the half-life of A1M in the blood is relatively short, several observations indicate that key functions of A1M, including reduction, heme binding, heme degradation and protection of red blood cells against hemolysis, are important intravascularly. Heme groups are present in various parts of the body, but mostly abundant in the blood as the oxygen binding molecule in Hb. If hemolysis occurs, extracellular Hb can oxidize nearby molecules and release its heme molecules causing further oxidative damage and trigger inflammation. A1M can counteract this in three ways: firstly, it stabilizes the red blood cells and protects them against various forms of stress to reduce the hemolytic event (Kristiansson et al., 2020a). Secondly, it can reduce oxidized extracellular Hb (Allhorn et al., 2005) to decrease its toxicity. Thirdly, A1M can bind and degrade free heme groups (Allhorn et al., 2002).

Another reported physiological function of A1M is to prevent MPO from oxidizing low density lipoprotein (LDL). Oxidation of LDL plays a central role in the pathogenesis of atherosclerosis (Heinecke, 1997), and MPO is one of the key mediators involved in the oxidation of LDL. A1M inhibits MPO from oxidizing LDL, and when interacting with MPO A1M is proteolytically cleaved, forming t-A1M (Cederlund et al., 2015). This suggests a role of A1M as a protector against oxidation of LDL, which may result in a decreased formation of atherosclerotic plaques.

### Extravascular

Extravascular A1M is often co-localized with structural components of the extracellular matrix (ECM), *c.f*. elastin and collagen (Bouic et al., 1985; Ødum and Nielsen, 1994; Olsson et al., 2011), and it has been shown to bind collagen *in vitro* (Santin and Cannas, 1999). A1M both inhibits the destruction of collagen fibrils exposed to Hb, heme or Fenton reaction-generated radicals, and repairs the collagen fibrils after the damage has already taken place (Olsson et al., 2011; Rutardottir et al., 2013). Additionally, perfusion of *ex vivo* placentas with A1M results in a large increase in the amount of collagen fibrils in the placenta (May et al., 2011). Moreover, evidence of ECM repair mechanisms has been found in studies on sheep, rabbits and mice with induced PE-like symptoms (Wester-Rosenlöf et al., 2014; Nääv et al., 2015; Erlandsson et al., 2019). Treatment with A1M resulted in reduced damage to structural components in the extravascular space of the kidney and placenta, including collagen fibrils. This suggests a biological role of A1M in protection and repair of the ECM and its structural components.

α_1_-microglobulin can bind to the surface of several different cell types, such as keratinocytes (Olsson et al., 2011), erythrocytes (Kristiansson et al., 2020a), neutrophils (Méndez et al., 1986), peripheral lymphocytes (Fernandez-Luna et al., 1988; Babiker-Mohamed et al., 1990b; Wester et al., 1998, 2000) and blood cell lines (Olsson et al., 2007, 2008). The phospholipid bilayer of the cell membrane is susceptible to oxidation by free radicals causing lipid peroxidation. The lipid peroxidation results in structural derangement of the membranes which alters the membrane fluidity, but can be inhibited by antioxidants such as vitamin C and E (Tai et al., 2010). The presence of A1M at the cell surface may enable both protection through radical scavenging and reduction of oxidation products formed in, or in the proximity of the cell membrane. Indeed, A1M has been found to protect a variety of cell types against oxidative stress-induced cell death, including keratinocytes (Olsson et al., 2011), erythroid cells (Olsson et al., 2008), erythrocytes (Kristiansson et al., 2020a), kidney cells (Kristiansson et al., 2020b) and liver cells (Olsson et al., 2010b; Rutardottir et al., 2013), supporting the proposed biological role as a protector against free radicals in the plasma membrane of cells.

### Intracellular

α_1_-microglobulin has been shown to bind to a subunit of Complex I of the mitochondria, which is a significant source of intracellular free radical production. This co-localization of A1M to Complex I was shown to preserve mitochondrial ATP-production during oxidative stress conditions (Olsson et al., 2013). The functional role of A1M in the mitochondria is not fully understood, but it has been suggested that A1M has a role in maintaining mitochondrial redox homeostasis (Olsson et al., 2013).

The co-synthesis of A1M and bikunin has been a mystery since its discovery over 30 years ago (Kaumeyer et al., 1986). Development of an A1M knockout mouse (A1M^–/–^) with a selective removal of the A1M exons and intact bikunin exons resulted in correct translation of signal peptide-containing bikunin but a defective synthesis of the high molecular weight forms of bikunin (Bergwik et al., 2020). The A1M^–/–^ mice showed signs of hepatic ER-stress, most likely due to misfolding of bikunin. A novel intracellular function of A1M as a chaperone during bikunin synthesis was therefore proposed (Bergwik et al., 2020).

## Conclusion

Approximately 45 years have passed since the discovery of A1M and since then new information about the protein has been published continuously. In this work, we have given an overview of the multifaceted functions of A1M and put them in a physiological relevant context. In brief, the lipocalin A1M employs three molecular mechanisms, chemical reductase activity, radical scavenging and heme binding to execute a physiological function as an antioxidant and tissue cleaning factor.

## Author Contributions

JB and BÅ: conceptualization and writing – finalization and submitting, and visualization. JB, AK, MA, MG, and BÅ: writing – original draft preparation and reviewing. AK, MG, and BÅ: funding acquisition. All authors have read and agreed to the published version of the manuscript.

## Conflict of Interest

BÅ and MG are co-founders and BÅ, MA, and MG are share-holders of Guard Therapeutics International AB, which holds the patent rights for medical uses of A1M. The remaining authors declare that the research was conducted in the absence of any commercial or financial relationships that could be construed as a potential conflict of interest.

## Footnotes

1 www.ncbi.nlm.nih.gov/gene

2 www.uniprot.org
